# Biocontrol of *Fusarium* and Other Fungal Diseases of Cereals Using Bacterial Compounds and Plant Extracts

**DOI:** 10.3390/molecules31101761

**Published:** 2026-05-20

**Authors:** Joanna Horoszkiewicz, Ewa Jajor, Marek Korbas, Jakub Danielewicz, Jan Bocianowski, Marzena Mikos-Szymańska, Tomasz Szymczak, Jagoda Kucharska, Monika Kobiałka, Marcin Podleśny

**Affiliations:** 1 Department of Mycology, Institute of Plant Protection—National Research Institute, Władysława Węgorka 20, 60-318 Poznan, Poland; e.jajor@iorpib.poznan.pl (E.J.); m.korbas@iorpib.poznan.pl (M.K.); j.danielewicz@iorpib.poznan.pl (J.D.); 2 Department of Mathematical and Statistical Methods, Poznań University of Life Sciences, Wojska Polskiego 28, 60-637 Poznan, Poland; jan.bocianowski@up.poznan.pl; 3 Grupa Azoty Zakłady Azotowe “Puławy” S.A., Al. Tysiąclecia Państwa Polskiego 13, 24-110 Pulawy, Poland; marzena.mikos-szymanska@grupaazoty.com (M.M.-S.); tomasz.szymczak@grupaazoty.com (T.S.); jagoda.kucharska@grupaazoty.com (J.K.); monika.kobialka2@grupaazoty.com (M.K.);; 4 Fermentation Technology Department, Institute of Agricultural and Food Biotechnology—State Research Institute, Rakowiecka 36, 02-532 Warszawa, Poland

**Keywords:** biological control, wheat protection, *Fusarium* spp.

## Abstract

Plant extracts and microbiological supernatants were subjected to qualitative and compositional analyses to characterize their bioactive profiles and assess their potential agricultural applications. The garlic (*Allium sativum*) extract was rich in allicin and selected free amino acids, contained betulin as the dominant triterpene, and displayed a favorable elemental profile with high levels of potassium, phosphorus, sulfur, calcium, and magnesium, with no detectable heavy metals. Detectable amounts of B-group vitamins and vitamin E isoforms were also identified. Qualitative phytochemical screening confirmed the presence of saponins and flavonoids in the garlic extract. The Jerusalem artichoke (*Helianthus tuberosus*) extract exhibited a significantly higher total phenolic content compared to the garlic extract, with qualitative analysis confirming the presence of saponins, tannins, and flavonoids, suggesting a broader spectrum of bioactive compounds. The two bacterial supernatants were characterized by HPLC analysis and differed in their metabolic profiles: the *Enterobacter* sp. fermentation broth contained glycerol, 2,3-butanediol, and acetic acid, while the *Paenibacillus* sp. supernatant additionally contained lactic acid, ethanol, and succinic acid, reflecting distinct fermentation pathways. The in vitro and greenhouse studies aimed to evaluate biological preparations for controlling wheat diseases caused by fungi of the *Fusarium* genus as well as diseases affecting the stem base. Plant extracts (garlic—*Allium sativum*, Jerusalem artichoke—*Helianthus tuberosus*) and supernatants (fermentation broths) obtained with the *Paenibacillus* and *Enterobacter* bacteria were tested at three concentrations. In laboratory experiments, the degree of inhibition of the growth of the mycelium of the tested fungal species was determined, while in greenhouse studies, the effectiveness in limiting the development of stem base diseases and the impact of the applied biopreparations on plant growth were evaluated. Among the plant extracts, *H. tuberosus* demonstrated superior antifungal activity, achieving up to 100% inhibition of *R. cerealis* mycelial growth at 10% concentration and reducing disease severity by 34.3% compared to the untreated control under greenhouse conditions. *Paenibacillus* sp. supernatant demonstrated strong in vitro antifungal activity. The results indicate that *H. tuberosus* extract represents a promising candidate for further field evaluation as a component of sustainable wheat protection programs.

## 1. Introduction

Modern sustainable agriculture requires biocontrol strategies that not only suppress plant pathogens but also promote plant health and growth. Traditional biocontrol evaluations focus primarily on pathogen inhibition, often overlooking the broader impacts on plant physiology and development. This narrow approach fails to capture the full potential of biological preparations that may simultaneously provide disease protection and growth enhancement through multiple mechanisms including induced systemic resistance, nutrient mobilization, and phytohormone production [1].

Wheat (*Triticum aestivum* L.) is one of the most important cereal crops globally, providing food security for billions of people. However, wheat production faces numerous challenges from fungal diseases, particularly those caused by *Fusarium* species, which can result in significant yield losses and mycotoxin contamination [2,3]. Traditional disease management relies heavily on synthetic fungicides, but their intensive use has led to environmental concerns, resistance development, and food safety issues [4]. *Rhizoctonia cerealis*, the causal agent of sharp eyespot in wheat, also poses a significant threat in wheat cultivation.

These challenges require a comprehensive approach that will determine the potential of biological alternatives in promoting plant health. The development of sustainable plant protection strategies has become a priority in modern agriculture. Biological control agents, including plant extracts and beneficial microorganisms, offer promising alternatives to chemical fungicides [5]. Plant-derived compounds possess diverse antimicrobial properties and often exhibit multiple modes of action, potentially reducing the risk of resistance development [6]. Understanding concentration–response relationships is crucial for practical implementation, as it enables optimization of application rates to balance efficacy with economic feasibility [7].

The development of biological alternatives to synthetic fungicides is increasingly urgent in the context of the progressive withdrawal of chemical active substances by the European Commission and growing concerns about environmental accumulation, resistance development, and food safety. Biological preparations, including plant extracts and fermentation-derived bacterial products, offer low environmental-risk alternatives that do not accumulate in soil, carry no mutagenic risk, and are compatible with integrated pest management (IPM) and organic farming frameworks. While synthetic fungicides such as prothioconazole provide high disease control efficacy [8,9,10], their exclusive use is unsustainable; biological preparations are therefore best positioned as complementary tools within a hybrid protection model.

Garlic (*Allium sativum* L.) contains sulfur compounds such as allicin, which demonstrate a broad-spectrum antimicrobial activity [11]. Garlic extracts showed a broad-spectrum fungicidal effect against a wide range of fungi [12]. However, the organosulfur compounds contained in garlic are very unstable and have low bioavailability, and their presence depends on how the garlic is processed when preparing garlic supplements [13].

Jerusalem artichoke (*Helianthus tuberosus* L.) produces various bioactive compounds including phenolic acids and flavonoids with antifungal properties [14]. Jerusalem artichoke is rich in inulin-type fructans, phenolic compounds, and sesquiterpene lactones that may contribute to both antifungal activity and plant growth stimulation through improved nutrient mobilization and stress tolerance mechanisms. Helianthus tuberosus tubers are rich in inulin, protein, and other bioactive components and are used to produce functional food ingredients. Many bioactive compounds have also been isolated from the aerial parts, which have antifungal, antioxidant, and anticancer properties [15].

Bacterial biocontrol agents, particularly *Paenibacillus* and *Enterobacter* species, can suppress plant pathogens through multiple mechanisms including antibiosis, competition, and induced systemic resistance [16]. Many *Paenibacillus* species can directly stimulate plant growth by fixing biological nitrogen, solubilizing phosphate, producing the phytohormone indole-3-acetic acid (IAA), and releasing siderophores that enable iron uptake. They can also provide protection against insects and pathogens, including bacteria, fungi, nematodes, and viruses. This is achieved by producing a variety of antimicrobials and insecticides and by inducing a hypersensitive plant defense response known as induced systemic resistance (ISR) [17]. Enterobacter has also been used to limit organisms of the genus *Pythium* on plant roots, such as those of cotton [6].

The present study was designed as a comparative evaluation of two contrasting plant-derived bioagent chemotypes: *Allium sativum*, which is primarily characterized by unstable organosulfur compounds (notably allicin), and *Helianthus tuberosus*, which is rich in polyphenols, flavonoids, inulin-type fructans, and sesquiterpene lactones. This dual-extract approach was motivated by the potential complementarity of their modes of antifungal action and by the objectives of the FERTI-UP research project, which encompassed both species as candidate biological inputs for sustainable crop protection. Importantly, the two extracts were evaluated independently rather than as a mixture, enabling separate assessment of their efficacy profiles across different pathogen species and concentration ranges.

The aim of the laboratory and greenhouse studies was to assess the antifungal effectiveness of selected plant extracts and bacterial biopreparations against the main fungal pathogens of wheat, in order to establish optimal dosage recommendations for sustainable wheat protection.

## 2. Results

The results presented below describe the qualitative and quantitative analysis of bioactive compounds identified in the examined extracts and supernatants. Moreover, the results show their antifungal effectiveness in laboratory and greenhouse studies.

### 2.1. Chemical Characterization of Plant Extracts

#### 2.1.1. Total Phenol Content

The results present the total phenolic content in the analyzed plant extracts, expressed as mg GAE/100 g of extract (gallic acid equivalents) (Table 1). The total phenolic content was significantly higher (*p* < 0.05) in *Helianthus tuberosus* (595.68 ± 15.42 mg GAE/100 g) compared to *Allium sativum* (507.24 ± 12.35 mg GAE/100 g). Since total phenolic content is often associated with antioxidant capacity, the higher value observed for Jerusalem artichoke may suggest a stronger potential antioxidant activity.

#### 2.1.2. Qualitative Phytochemical Analysis

Qualitative analyses of the plant extracts were conducted to determine the presence of selected groups of bioactive compounds, including alkaloids, flavonoids, tannins, saponins, coumarins, and quinones.

The results of the phytochemical screening revealed differences in the composition of the analyzed extracts. In the garlic extract (*Allium sativum*), the presence of saponins and flavonoids was confirmed. In the Jerusalem artichoke extract (*Helianthus tuberosus*), saponins, tannins, and flavonoids were detected (Table 2).

Extended analyses are presented only for the garlic extract, as this extract was selected for further evaluation in subsequent stages of the project.

The garlic extract contained 6.45 µg/mL of abscisic acid (ABA) (Table 3). Elemental composition revealed high levels of potassium (2831 mg/L), phosphorus (860 mg/L), sulfur (482 mg/L), calcium (348 mg/L), and magnesium (128 mg/L), while heavy metals (Hg, Cr, Ni, Pb, Co) were below the detection limit (Table 4).

The garlic extract was characterized by a high content of allicin (83.29 mg/L), the main sulfur-containing bioactive compound (Table 5). Among the amino acids, L-aspartic acid was the most abundant (230 mg/L), followed by L-threonine (57 mg/L) and L-asparagine (10.44 mg/L), while L-cysteine was below the minimum quantification limit. In the group of triterpenes, betulin was present at a considerable concentration (275.10 mg/L), whereas oleanolic acid, maslinic acid, and lupeol were not detected above the quantification limit. All polyphenols were below the limit of quantification. Regarding vitamins, B-group vitamins were detected at low levels: B2 at 0.41 mg/L, B3 at 0.64 mg/L, and B6 at 1.06 mg/L, while folic acid (B9), vitamin C, and vitamin A were below the detection limit. Among vitamin E compounds, D-α-tocotrienol and D-γ-tocopherol were the main components, present at 28.02 mg/L and 24.29 mg/L, respectively.

### 2.2. Chemical Characterization of Microbiological Supernatants

A validation of the HPLC method used in the quantification of glycerol and fermentation products is presented in Table 6.

Data of each analyte were generated in triplicate (total number of points = 12 for each analyte). Injection volume was 10 µL in all runs.

**Figure 1:**
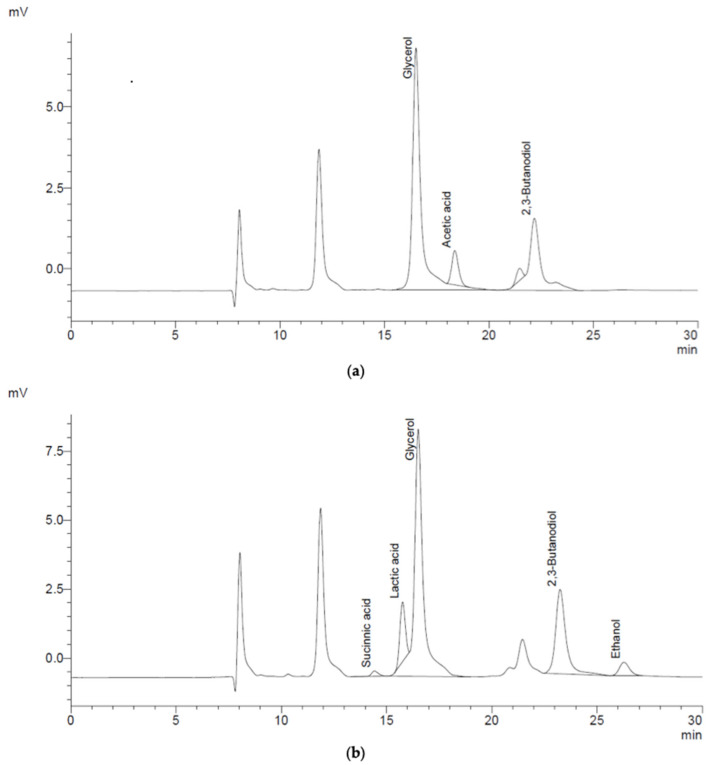
(**a**) HPLC profiles (refraction index detector) for the supernatant obtained with the *Enterobacter* spp. bacteria. (**b**) HPLC profiles (refraction index detector) for the supernatant obtained with the *Paenibacillus* spp. bacteria.

As shown in Figure 1a, the supernatant obtained with the *Enterobacter* spp. contains acetic acid (1.19 g/L), 2,3-butanodiol (2.55 g/L) and glycerol (6.29 g/L). Moreover, as shown in Figure 1b, the supernatant obtained with the *Paenibacillus* spp. contains succinic acid (0.14 g/L), lactic acid (1.35 g/L), ethanol (1.01 g/L), 2,3-butanodiol (2.85 g/L) and glycerol (7.85 g/L). All products present in both supernatants were based on the HPLC method (see Table 6).

### 2.3. Experiment In Vitro

The results of the analysis of variance indicate a statistically significant influence of both factors and their interactions on the values of the observed traits for all pathogens. The plant extracts from Jerusalem artichoke and garlic, as well as post-fermentation broths obtained using bacteria of the genera *Paenibacillus* and *Enterobacter*, showed varied efficacy in inhibiting the mycelial growth of the studied fungal species. This depended on the type of biopreparation used, its concentration, and the fungal species (Table 7).

The addition of garlic extract to PDA medium resulted in more than 50% inhibition of mycelial growth in three of the studied fungal species (Table 7). The inhibition of mycelial growth of *F. poae* and *R. cerealis* following the application of garlic extract at concentrations of 5 and 10% amounted to 55.9 and 62.4%, and 65.7 and 89.1%, respectively. Considerable inhibition of mycelial growth of *T. yallundae* was also obtained—72.2% at a 10% concentration of garlic extract. The addition of the second tested plant extract, derived from Jerusalem artichoke, demonstrated greater efficacy compared to garlic extract. High efficacy of mycelial growth inhibition of *R. cerealis* was recorded at all concentrations—75% inhibition at 1% concentration and 100% inhibition at 10% concentration. Inhibition of mycelial growth exceeding 50% was also recorded for Fusarium species, including *F. culmorum*, *F. poae*, and *F. graminearum*. At a 10% concentration, inhibition of mycelial growth of *F. culmorum* amounted to 74.6%, and that of *F. graminearum* to 67.4%. At the same concentration, growth of *T. yallundae* was inhibited by 56.9%.

Among the tested post-fermentation broths, greater efficacy of mycelial growth inhibition across all tested concentrations was observed following the application of the Paenibacillus-derived broth (Table 7). Inhibition of mycelial growth of the tested fungal isolates exceeded 50% (with the exception of the broth at 5% concentration in limiting the growth of *F. culmorum*—49.3%). Only the application of the Enterobacter-derived broth at all concentrations reduced mycelial growth of *T. yallundae* by more than 50% (ranging from 61.2 to 73.8%), and at a concentration of 1% also inhibited the growth of *F. avenaceum* by 59.1%. For *F. fujikuroi*, mycelial growth inhibition amounted to 53.5% following the application of the 5% post-fermentation broth obtained using *Enterobacter* bacteria.

The application of the synthetic fungicide as a reference agent, containing prothioconazole as the active substance, demonstrated 100% efficacy in inhibiting mycelial growth of all studied fungal species.

**Figure 2:**
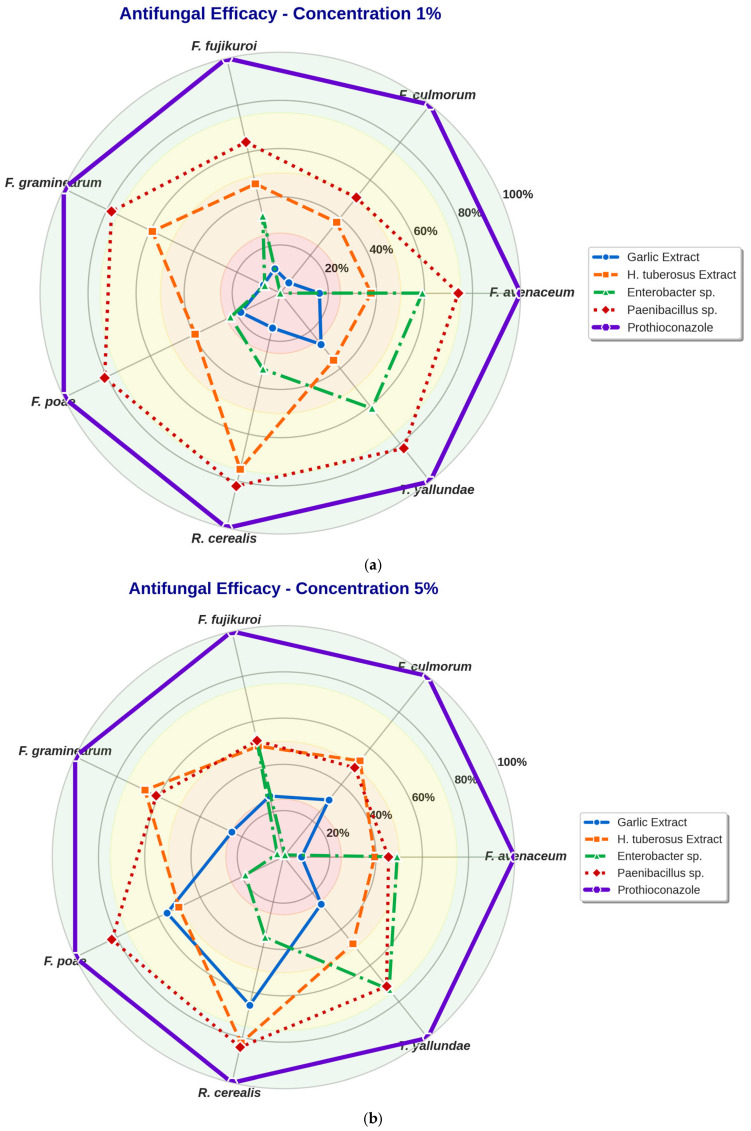

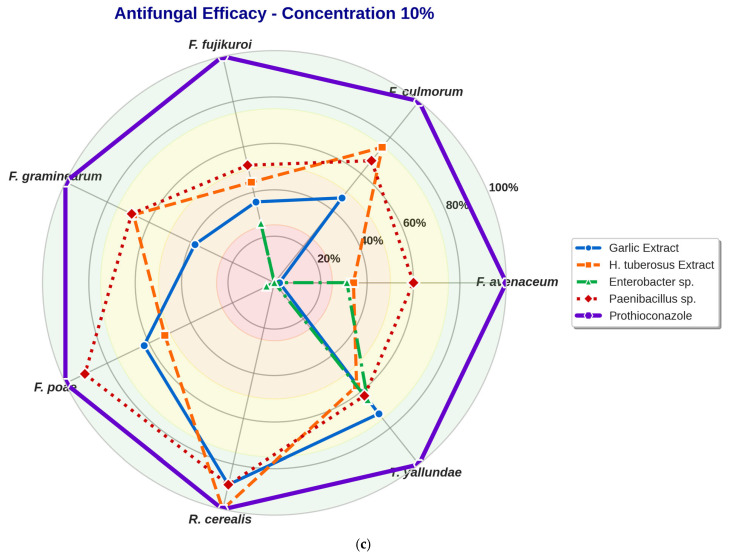
The antifungal efficacy (% inhibition of mycelial growth) of biocontrol agents at different concentrations against seven wheat fungal pathogens: (**a**) at 1%, (**b**) at 5%, (**c**) at 10%.

Analyzing the effectiveness of the concentrations of plant extracts and post-fermentation broths used in the experiment, their effectiveness was compiled in the form of radar charts (Figure 2a–c).

Distinct concentration–response (Figure 2a) patterns were observed for different pathogen-biocontrol agent combinations. *H. tuberosus* extract showed strong efficacy even at low concentrations against certain pathogens, particularly *R. cerealis* (75.0% inhibition at 1%), suggesting potent bioactive compounds with low minimum inhibitory concentrations.

Garlic extract exhibited variable concentration responses, with some pathogens showing improved efficacy at higher concentrations (*F. poae*, *T. yallundae*), while others demonstrated decreased efficacy (*F. avenaceum*), possibly due to concentration-dependent changes in allicin stability or antagonistic effects of multiple sulfur compounds.

### 2.4. Greenhouse Experiment

Complete plant health assessments revealed significant differences in both disease control and biomass allocation patterns among treatments (Table 8). The comprehensive analysis demonstrated that biological preparations not only provided pathogen suppression but also optimized plant resource allocation, promoting efficient above-ground growth while reducing root biomass investment typical of stress responses.

*H. tuberosus* extract and supernatant (fermentation broth) obtained with the *Enterobacter* sp. bacteria achieved significant disease control under greenhouse conditions, reducing disease severity by 34.3% and 32.8%, respectively, compared to untreated controls (*p* < 0.01). While not matching the exceptional efficacy of prothioconazole (78.2% reduction), these biological treatments provided commercially meaningful disease suppression levels. *H. tuberosus* extract emerged as a superior dual-action biological preparation providing both disease control and growth promotion (13% shoot growth increase, 17% biomass increase). *H. tuberosus* extract promoted shoot elongation (43.7 cm vs. 38.6 cm control, *p* < 0.05) and increased fresh weight production (36.9 g vs. 31.5 g control, *p* < 0.05), indicating growth-promoting activities beyond direct pathogen suppression. Prothioconazole enhanced root development (22.4 cm vs. 22.0 cm control) and biomass accumulation, demonstrating that effective disease control can indirectly promote plant growth through stress reduction.

Root fresh weight analysis revealed striking differences in resource allocation strategies among treatments. Untreated controls exhibited the highest root biomass (9.1 ± 3.2 g), indicating typical stress response allocation toward root development for enhanced nutrient and water acquisition under pathogen pressure. In contrast, all biological treatments significantly reduced root biomass investment (4.7–5.2 g, *p* < 0.01), with *Enterobacter* sp. achieving the lowest root biomass (4.7 ± 0.6 g, *p* < 0.001), demonstrating the most efficient resource allocation strategy.

Growth allocation efficiency analysis revealed that successful biological treatments achieved superior shoot-to-root biomass ratios compared to stressed controls. *Enterobacter* sp. and *H. tuberosus* extract both achieved optimal efficiency ratios of 7.1, indicating balanced resource allocation toward productive shoot growth while maintaining adequate root function. This contrasts sharply with the inefficient control allocation (ratio 3.5) typical of pathogen-stressed plants investing disproportionately in root development.

**Figure 3:**
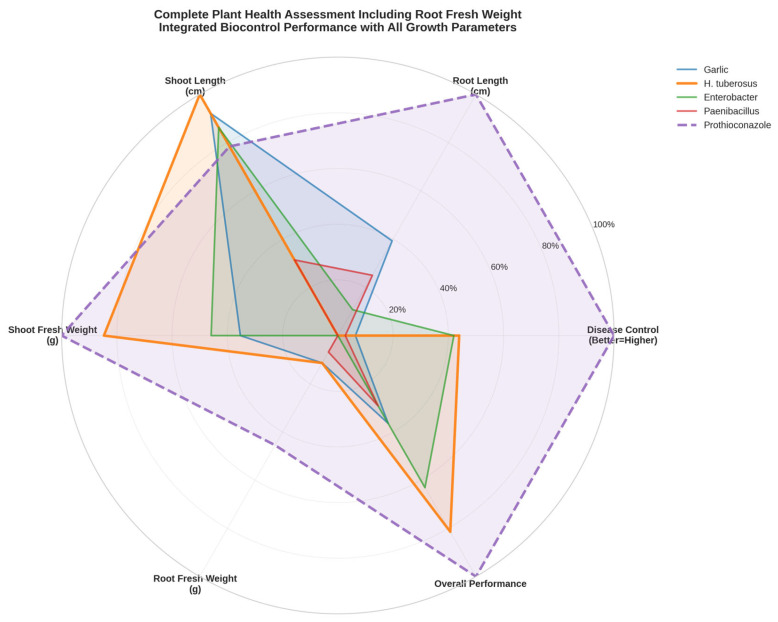
Complete plant assessment radar profiles incorporating all parameters.

Complete plant health assessment incorporating all six measured parameters revealed distinct multi-dimensional performance signatures for each biological preparation (Figure 3). The comprehensive radar profiling approach, including root fresh weight analysis, provides unprecedented insights into the holistic effects of biocontrol treatments on plant resource allocation and overall health status.

*H. tuberosus* extract demonstrated the most balanced and expansive radar profile, excelling particularly in disease control, shoot development, and overall performance while maintaining efficient root biomass allocation. *Enterobacter* sp. showed a specialized signature with exceptional root efficiency (minimal root fresh weight) combined with strong disease control, indicating highly optimized resource allocation strategies.

Prothioconazole exhibited the most expansive overall profile, particularly in disease control and total biomass production, but with less efficient root-shoot allocation compared to optimized biological treatments. The chemical reference achieved maximum disease suppression but at the cost of balanced resource allocation, suggesting that biological preparations may offer more sustainable plant health optimization strategies.

## 3. Discussion

This study focused on targeted and screening-level chemical characterization rather than full structural elucidation of isolated compounds.

In the present study, aqueous extracts obtained from *Allium sativum* (garlic) and Helianthus tuberosus (Jerusalem artichoke) leaves were evaluated for their total phenolic content (TPC) in the initial solutions obtained after extraction. The garlic extract exhibited a TPC of 507.24 mg/100 g of extract, whereas the extract obtained from Jerusalem artichoke leaves showed a higher value of 595.68 mg/100 g of extract. In the present study, the relatively high total phenolic content was observed in extracts obtained from *Allium sativum* (garlic) and Jerusalem artichoke leaves may have contributed to the reduction in plant diseases observed in the pot experiments. The total phenolic content of the garlic extract obtained in the present study (507.24 mg/100 g) is comparable with values reported in the literature. For example, aged garlic extract was reported to contain approximately 562.6 mg GAE/100 g [18]. Similarly, other studies indicate that the total phenolic content of garlic may range between 3.4 and 10.8 mg GAE/g of dry matter depending on cultivar and growing conditions [19]. According to Showkat et al. [20], phenolic compounds in Jerusalem artichoke leaves may reach 4.5–5.7 mg GAE/g dry matter.

Flavonoids and tannins are known for their antioxidant properties, while saponins exhibit antimicrobial and anti-inflammatory potential. The compounds detected in plant extracts belong to groups widely recognized for their biological activity. The broader spectrum of identified phytochemicals in Jerusalem artichoke may suggest a diverse biological activity profile. However, since the analysis was qualitative, it confirms only the presence or absence of specific compound groups and does not provide information about their concentration. The results of the qualitative phytochemical analysis demonstrated the presence of flavonoids, tannins, and saponins in the investigated plant extracts. These findings are consistent with previous literature reports describing the phytochemical profiles of the studied species.

Garlic (*Allium sativum*) is widely recognized for its rich content of sulfur-containing compounds, particularly allicin. However, studies have also confirmed the presence of flavonoids and saponins in garlic extracts. According to Phytochemical Methods: A Guide to Modern Techniques of Plant Analysis, flavonoids are commonly distributed among higher plants and contribute significantly to antioxidant capacity. The presence of flavonoids in garlic extract observed in this study is therefore consistent with earlier phytochemical screenings. The results indicate that the garlic extract is rich in sulfur-containing compounds (allicin), certain amino acids, betulin, and vitamin E, which are known for their bioactive properties. The low or undetectable levels of polyphenols, vitamin C, and A suggest selective preservation or extraction efficiency. High concentrations of essential minerals combined with negligible heavy metals highlight the nutritional and safety profile of the extract. Overall, these findings support the extract’s potential as a functional ingredient with health-promoting properties.

Jerusalem artichoke has been reported to contain phenolic acids, flavonoids, and tannins, which are associated with antioxidant and antimicrobial activities. Literature data indicate that members of the Asteraceae family are often rich in polyphenolic compounds. The broader spectrum of phytochemicals identified in Jerusalem artichoke in the present study may therefore explain its relatively high total phenolic content observed in quantitative analysis and suggests potential multifunctional biological activity.

It should be emphasized that qualitative phytochemical screening provides preliminary information only. As noted in standard pharmacognostic methodologies, these assays confirm the presence or absence of selected groups of secondary metabolites but do not allow for precise quantification. Therefore, further studies involving chromatographic techniques such as HPLC or GC–MS would be necessary to identify and quantify individual bioactive constituents.

Regarding supernatants, the obtained results demonstrate clear differences in the metabolic profiles of *Enterobacter* spp. and *Paenibacillus* spp. The analysis of the supernatants indicates that both microorganisms are capable of producing 2,3-butanediol and glycerol, although in different amounts, suggesting variations in carbon flux distribution and metabolic regulation.

The progressive withdrawal of numerous active substances by the European Commission in recent years serves as a compelling impetus for research endeavors aimed at broadening the available strategies for crop protection and pest management. The main reduction in active substances is not only due to a falling-off of chemicals but also a significant decay of BCA impacting crop protection with a small increase in fungicides with a high replacement rate [21]. The supplementation and/or replacement of synthetic compounds with biopreparations aligns with the hybrid protection model, which combines, among other approaches, the use of chemical and biological methods. The conducted in vitro and greenhouse studies allowed for the identification of research combinations for further field experiments, which may contribute to determining the potential for their application in broadly understood agricultural practice.

Distinct concentration–response patterns were observed for different pathogen-biocontrol agent combinations. Supernatant obtained with the *Paenibacillus* demonstrated the most consistent dose–response relationships across selected Fusarium species (*F. poae*) and *R. cerealis*, with linear or near-linear increases in efficacy from 1% (*w*/*v*) to 10% (*w*/*v*) concentrations. In studies of Horoszkiewicz et al. [22] investigated the inhibition of mycelial growth of Fusarium fungi occurring in maize. Among the tested products, microbiological extracts, based on supernatant obtained using bacteria of the genus *Paenibacillus*, proved to be the most effective in limiting the growth of fungal mycelium. In the same study, the supernatant obtained with *Paenibacillus* supernatant at concentrations of 1 and 2% (*w*/*v*) had a positive effect on the germination index of maize seeds compared to the control (distilled water), and is therefore considered to possess biostimulant activity. The literature indicates the usefulness of numerous organisms, such as *Pseudomonas*, *Pasteuria*, *Talaromyces*, *Trichoderma* spp., *Bacillus subtilis*, and *Pseudomonas* spp., which have been used as biocontrol agents to protect plants against soil-borne wheat diseases, including sharp eyespot in the field [23].

The moderate plant health effects observed with *Paenibacillus* sp. despite strong antifungal efficacy suggest that this preparation functions primarily through direct pathogen suppression rather than plant growth promotion. This finding is consistent with the known antibiotic production capabilities of *Paenibacillus* species, which may provide excellent pathogen control but limited growth enhancement effects [17]. In greenhouse experiments, among the tested supernatants, a lower incidence of seedling blight was observed following the application of the supernatant obtained with Enterobacter. The use of this supernatant was also reflected in the growth efficiency index, which amounted to 7.1. The same result was recorded following the application of the *H. tuberosus* extract.

The application of *H. tuberosus* extract, even against strong pathogens such as *R. cerealis* and Fusarium species, demonstrated notable bioactive effects. The inhibitory properties observed indicate that Jerusalem artichoke possesses pathogen-suppressing activity attributed to its content of phenolic compounds and flavonoids. *H. tuberosus* extract thus represents a promising biological preparation with potential for application in both disease control and growth stimulation.

The discovery that *H. tuberosus* extract simultaneously provides disease control and growth promotion represents a significant advancement in biocontrol science, challenging the traditional view of biocontrol agents as primarily pathogen-suppressive tools. This dual-action profile is not paradoxical but rather reflects the multifunctional biological activity of the diverse phytochemical matrix present in Jerusalem artichoke leaves. Three non-mutually exclusive mechanisms may explain the observed growth promotion. First, inulin-type fructans function as prebiotics in the rhizosphere, selectively stimulating beneficial microorganisms that enhance nutrient mobilization. Polyphenolic compounds (including quercetin glycosides and caffeic acid derivatives) reduce oxidative stress under pathogen pressure, preserving photosynthetic capacity. Sesquiterpene lactones characteristic of the *Asteraceae* family may exhibit auxin-like activity, potentially stimulating cell elongation. The concurrent suppression of *R. cerealis* and *Fusarium* spp. combined with active growth promotion through these phytochemical mechanisms likely acts synergistically, explaining the superior biomass accumulation observed in treated plants. This exceptional performance in both disease control and biomass production suggests the presence of bioactive compounds with true dual functionality, highlighting the importance of holistic multi-parameter plant health assessment approaches [15,24,25,26,27,28].

A striking pattern observed in the results connected to biometric measures needs to be clarified. The root fresh weight of pathogen-inoculated untreated control plants (9.1 ± 3.2 g) was substantially and significantly higher than that of all treated groups (4.7–6.7 g, *p* < 0.01). This phenomenon reflects a well-documented plant stress response known as stress-induced compensatory root proliferation [29]. Under fungal infection pressure—particularly from Fusarium species causing stem base diseases—plants reallocate photosynthate resources toward root system expansion as a compensatory mechanism to maintain adequate water and nutrient uptake capacity when root function is impaired. This stress-driven resource allocation is inherently inefficient, resulting in elevated root biomass at the expense of productive shoot growth. Biological and chemical treatments that successfully suppressed pathogen activity eliminated this stress signal, allowing plants to redirect resources from root proliferation toward shoot development, as evidenced by the markedly improved shoot-to-root fresh weight ratios (Growth Efficiency index: 5.9–7.1 for treated groups vs. 3.5 for the untreated control). The consistently low root fresh weight across all treatment groups—including prothioconazole—confirms that effective disease suppression, regardless of the agent type, restores normal biomass allocation patterns. This interpretation is consistent with reports on compensatory root architecture plasticity under biotic stress, where pathogen-free conditions achieved through any protective treatment normalize the root/shoot allocation ratio [30,31,32].

Garlic extract exhibited variable concentration responses, with some pathogens showing improved efficacy at higher concentrations (*F. graminearum*, *F. poae*, *F. fujikuroi*), while others demonstrated decreased efficacy (*F. avenaceum*), possibly due to concentration-dependent changes in allicin stability or antagonistic effects of multiple sulfur compounds.

The garlic extract, similarly to the supernatant obtained with *Paenibacillus* bacteria, did not contribute to the reduction in seedling blight development. Numerous authors indicate the usefulness of garlic extract or commercially available biopreparations. In the study by Panasiewicz et al. [33], the utility of garlic extract for seed dressing of winter wheat was demonstrated. A positive effect of the preparation on root length, plant vigor, and growth rate was observed, with the exception of root number. However, such a positive effect was not obtained for all investigated cereal species. In the experiment conducted by Islam [34], garlic extract used for wheat seed dressing most effectively reduced, among the tested extracts including marigold, neem, and allamanda, the development of pathogenic fungi, including those of the genus Fusarium. In the studies by Baturo [35] and Horoszkiewicz-Janka and Jajor [36], seed dressing of winter barley and wheat, respectively, with garlic extract yielded a positive effect on plant health compared to the control. In other studies by Horoszkiewicz-Janka et al. [37], in which oat grain was dressed with garlic extract, improved emergence and health of emerging plants were obtained. In the present study, in which garlic extract was applied as a foliar spray, no significant effect on the incidence of seedling blight in the early developmental stages of wheat was observed.

These concentration–response data provide a scientific foundation for developing economically viable biological control programs in wheat production. The identification of minimum effective concentrations and optimal dosage ranges for specific pathogen targets represents a critical advancement toward practical implementation of sustainable plant protection strategies. Future research should focus on field validation of these laboratory-derived concentration recommendations and integration with existing disease management practices.

Translating the laboratory and greenhouse findings reported here into field-scale implementation will require addressing several practical challenges. The production of standardized plant extracts at agricultural scale demands consistent raw material quality and validated extraction protocols; however, *H. tuberosus* is already cultivated industrially for inulin production, providing an accessible and economically scalable source. Bacterial supernatants require bioreactor fermentation infrastructure and temperature-controlled supply chains, conditions already standard in commercial biocontrol product manufacturing. Both preparation types are physically compatible with existing agricultural spray equipment at the concentrations tested. The primary regulatory pathway—biostimulant registration under EU Regulation 2019/1009 or biocontrol product registration under plant protection product legislation—represents the most significant implementation bottleneck and will require field-scale efficacy and safety data. The progressive withdrawal of synthetic active substances by the European Commission, combined with increasing demand for residue-free and organically certified crop production, creates a favorable regulatory and market context for accelerating the transition of these preparations toward commercial application. Also the adaptation of the Common Agricultural Policy to the conditions of the EU–Mercosur Agreement will constitute a significant challenge in adjusting agricultural production inputs to evolving requirements [38,39,40].

## 4. Materials and Methods

### 4.1. Plant Extract Preparation

Plant extracts of *Allium sativum* and *Helianthus tuberosus were obtained by* ultrasound-assisted extraction (USE). For this purpose, a sonicator (Vibra Cell, SONICS & Materials, Inc. Newtown, CT, USA) with an installed probe (209-C) and a magnetic stirrer (HeiTec, Heidolph MR, NG Zeist, The Netherlands) set in the range from 130 to 500 rpm, were used. The applied extraction conditions were preliminary and selected for screening purposes. Optimization of extraction parameters was conducted at a later stage of the project and is beyond the scope of the present study. No alternative extraction methods were evaluated for these extracts within the scope of this study. The extraction conditions are shown in Table 9. The prepared extracts were subsequently diluted to final concentrations of 1% (*w*/*v*) and 10% (*w*/*v*), corresponding to supernatants and plant extracts, respectively. Due to differences in drug-extract ratios, the nominal extract concentrations (% *w*/*v*) do not correspond to equal absolute contents of bioactive compounds; therefore, the results should be interpreted in terms of biological response rather than strict chemical equivalence.

### 4.2. Supernatant Preparation

Fermentation broths (supernatants) were obtained from bioreactor cultures of bacteria belonging to the genera *Enterobacter* and *Paenibacillus*. The cultivation was carried out under controlled conditions, including regulation of temperature, pH, and agitation, to ensure optimal microbial growth and metabolite production (Table 9).

### 4.3. Chemical Characterization of the Plant Extracts and Microbial Supernatants

Chemical characterization was performed using spectrophotometric assays, qualitative phytochemical screening, and targeted chromatographic and mass-spectrometric analyses, enabling identification of major bioactive constituents within a screening framework. All applied analytical procedures were designed for chemical characterization and screening of bioactive components rather than complete structural elucidation of isolated compounds.

### 4.4. Determination of Total Phenol Content (TPC)

Total phenol content (TPC) in the extracts of *Allium sativum* and *Helianthus tuberosus* was determined by method using the Folin–Ciocalteu reagent (Table 9).

- preparation of the standard curve

In order to prepare the standard curve, a working solution of gallic acid with a concentration of 2 g/L in distilled water was prepared. Specified volumes of the working solution were taken into 50 mL volumetric flasks, and the flasks were filled to the mark. From each concentration, 0.5 mL of the gallic acid standard solution was taken into 10 mL volumetric flasks. To each flask, 4 mL of distilled water and 0.5 mL of Folin–Ciocalteu reagent were added and mixed. After 2 min from the addition of the FC reagent, 2 mL of 20% Na_2_CO_3_ solution was added, the flasks were filled to the mark with distilled water, and incubated at 40 °C for 30 min. After this time, the absorbance was measured at a wavelength of λ = 760 nm, using water as the reference. Analyses were performed in triplicate.

- sample preparation for determination

The determination was carried out by sequentially adding to 10 mL flasks: 4 mL of distilled water, 0.5 mL of the tested preparation, 0.5 mL of the FC reagent, mixed, and after 2 min, 0.5 mL of 20% sodium carbonate was added and topped up with distilled water to the mark. The samples were then incubated at 40 °C for 30 min, after which the absorbances were measured. Measurements were performed against a reference sample in which the tested extracts were replaced with 0.5 mL of water (7.5 mL of water + 0.5 mL FC + 2 mL of 20% Na_2_CO_3_). Analyses were performed in three replications.

### 4.5. Qualitative Phytochemical Analysis

The qualitative phytochemical screening of the plant extracts was performed to detect the presence of major classes of secondary metabolites, including saponins, tannins, flavonoids, terpenoids, anthraquinones, cardiac glycosides, coumarins, phlobatannins, and carbohydrates. Standard procedures described in the literature were applied.

Saponins—Foam Test: 5 mL of the extract was mixed with 5 mL distilled water in a test tube, sealed, and shaken vigorously for 10 s to generate foam. The samples were left for 10 min, after which 4 drops of 1 M HCl were added. The formation of stable foam (1–10 cm) for at least 10 min indicated the presence of saponins.

Saponins—Olive Oil Test: 10 mL of extract was mixed with distilled water and boiled if necessary. Ten drops of olive oil were added and mixed for 10 min using a roller mixer at 220 rpm. The formation of a stable emulsion indicated the presence of saponins.

Tannins: 1–2 mL of extract was mixed with a few drops of 1% FeSO_4_ solution. Formation of a green or black precipitate indicated the presence of tannins.

Terpenoids—Salkowski Test: 2 mL chloroform and 5 mL extract were mixed in a test tube, followed by careful addition of 3 mL concentrated H_2_SO_4_. The appearance of a reddish-brown coloration at the interface confirmed the presence of terpenoids.

Anthraquinones—Borntrager’s Test: 3 mL aqueous extract was shaken with 3 mL benzene, filtered, and 5 mL 10% ammonia solution was added. The appearance of pink, red, or violet coloration in the ammonia layer indicated the presence of free anthraquinones.

Cardiac Glycosides—Keller–Killiani Test: 5 mL extract was mixed with 2 mL cold acetic acid, one drop of FeCl_3_, and 1 mL concentrated H_2_SO_4_. A brown ring at the interface indicated the presence of cardiac glycosides.

Flavonoids: 10 mL extract was mixed with 5 mL diluted ammonia solution, followed by a few drops of concentrated H_2_SO_4_. The appearance of a yellow color indicated the presence of flavonoids.

Carbohydrates: 3 mL aqueous extract was mixed with ~1 mL iodine solution. Violet coloration at the interface indicated the presence of carbohydrates.

Coumarins: 5 mL extract was placed in a test tube covered with filter paper soaked in 1 M NaOH and heated in a water bath. Yellow fluorescence under UV light indicated the presence of coumarins.

Phlobatannins: 200 mg extract was boiled in 6 mL 1% HCl. Formation of a red precipitate indicated the presence of phlobatannins.

### 4.6. Determination of Abscisic Acid (ABA)

Filtrates were analyzed for ABA concentration using the Agrisera ELISA kit (Agrisera AB, Vännäs, Sweden), following the manufacturer’s instructions. Both undiluted and 1:2 diluted samples were tested; undiluted samples gave false results due to antigen saturation; so, only diluted samples were used. ABA concentrations were calculated from a logistic standard curve generated with the provided standards.

### 4.7. Determination of Selected Element Contents

Elemental analysis was performed after sample mineralization in 65% nitric acid (5 mL sample + 5 mL acid) using a microwave digestion system (EthosUp, Milestone, Italy). Digests were quantitatively transferred to 50 mL Falcon tubes and diluted with deionized water to 50 mL. The elemental composition of the solutions was analyzed using ICP-OES (Avio 200, Perkin Elmer, Shelton, CT, USA).

### 4.8. Chromatographic Analyses of Garlic Extract

Chromatographic determinations were performed using UHPLC-DAD, UHPLC-FLD (Dionex Ultimate 3000, Agilent 1260, (Agilent Technologies, Santa Clara, CA, USA) and LC-ESI-MS/MS (Shimadzu LCMS-8045 Agilent Technologies, Santa Clara, CA, USA).

Analytes: UHPLC-DAD: allicin, vitamin C, triterpenes (oleanolic acid, maslinic acid, lupeol, betulin), amygdalin; UHPLC-FLD: vitamins A and E (tocopherols and tocotrienols); LC-ESI-MS/MS: B vitamins, free amino acids, polyphenols.

Sample Preparation: Extracts were diluted with appropriate solvents, sonicated, filtered (0.22–0.45 µm), and injected in duplicate. Solvent blanks were included.

Chromatographic Conditions (summary): Columns: C18-based UHPLC columns (Hypersil GOLD, Luna Omega, Syncronics C18, Discovery HS F5, Milwaukee, WI, USA), Mobile phases: water/acid, methanol, acetonitrile mixtures depending on analyte; Flow rates: 0.15–0.64 mL/min, isocratic or gradient programs; Column temperatures: 20–50 °C; autosampler: 10–20 °C; Detection: DAD (205–254 nm), FLD (EX/EM 295–329/330–472 nm), MS/MS MRM (positive or negative mode).

#### Determination of Allicin Concentration

Allicin concentration was quantified by UHPLC-DAD using an external standard method. Quantification was based on peak areas recorded at 254 ± 4 nm and calculated using a calibration curve prepared from a commercially available allicin standard dissolved in methanol.

Due to the instability of allicin, sample preparation and analysis were performed under controlled conditions to minimize degradation. The extract was diluted in methanol, sonicated for 10 min, immediately filtered, and analyzed without storage. The autosampler temperature was set to 20 °C to reduce potential thermal degradation. Each sample was analyzed in duplicate, and a solvent blank was included.

### 4.9. Chromatographic Analysis of Supernatants

Samples for the detection of by-products were prepared by centrifuging the culture broth at 9000× *g* for 5 min. After dilution with water (1:3), the resulting supernatant was analysed using a high-performance liquid chromatography system (Prominence, Shimadzu, Nakagyo-ku, Kyoto, Japan) equipped with an anion exchange column (Aminex HPX-87H, Bio-Rad, Hercules, CA, USA) and a refractive index detector (Shimadzu RID 20A, Agilent Technologies, Santa Clara, CA, USA). The mobile phase was 0.03 M sulfuric acid at 50 °C [41].

### 4.10. The Antifungal Efficacy

The antifungal efficacy evaluation followed a factorial completely randomized design with three factors: biocontrol treatments (five levels), concentrations (three levels), and fungal pathogens (seven species). Each treatment combination was replicated six times (n = 6), resulting in 630 experimental units. The response variable was percentage inhibition of mycelial growth calculated as follows:

Inhibition (%) = [(Control diameter − Treatment diameter)/Control diameter] × 100.

Prior to analysis, data were examined for outliers using the interquartile range (IQR) method, where values exceeding Q_3_ + 1.5(IQR) or below Q_1_—1.5(IQR) were investigated. Normality was assessed using the Shapiro–Wilk test for each pathogen-treatment combination. Homogeneity of variance was evaluated using Levene’s test. When assumptions were violated, data were subjected to arcsine square-root transformation:

$$Transformed\;value=arcsin\left(\sqrt{\frac{percentage}{100}}\right)$$

For each fungal pathogen, a two-way ANOVA was conducted using the linear model:

Y*_ijk_* = μ + T*_i_* + C*_j_* + (TC)*_ij_* + ε*_ijk_*,

where Y*_ijk_* represents the efficacy value for the *i*-th treatment, *j*-th concentration, and *k*-th replicate; μ is the grand mean; T*_i_* is the effect of the *i*-th biocontrol treatment; C*_j_* is the effect of the *j*-th concentration; (TC)*_ij_* is the interaction between treatment and concentration; and ε*_ijk_* is the random error term. When ANOVA indicated significant differences (*p* < 0.05), Tukey’s Honestly Significant Difference (HSD) post hoc test was applied to control the family-wise error rate at α = 0.05. The Tukey HSD test was selected for its balanced control of Type I and Type II errors and appropriateness for all pairwise comparisons among treatments within each concentration level.

Linear regression analysis was performed for each treatment-pathogen combination to evaluate dose–response relationships:

Efficacy = β_0_ + β_1_(Concentration) + ε

where β_0_ is the intercept, β_1_ is the slope coefficient, and ε is the error term.

### 4.11. Laboratory and Greenhouse Studies

Two types of experiments were conducted with selected preparations of plant and bacterial origin—laboratory and greenhouse studies.

In the laboratory experiment, mycelial growth and the percentage of growth inhibition were assessed following the application of biopreparations compared to a synthetic fungicide containing prothioconazole. The untreated combination consisted of objects without any additives. The bio compounds used in the experiment (in vitro and greenhouse) and the method of their preparation are presented in Table 9. The tested bioproducts were applied at three concentrations, while the dose of the reference fungicide was calculated per hectare (per 200 L of water). The pathogens tested in the experiment are fungi responsible for, among others, *Fusarium* foot and root rot, eyespot of cereals, and sharp eyespot of cereals. The isolates of the studied fungi are part of the collection maintained at the Department of Mycology of the Institute of Plant Protection—National Research Institute. The isolates used in the in vitro experiment are listed in Table 10. Plant material was collected from the winter wheat fields in the Wielkopolska and Lubuskie provinces in order to obtain the studied fungi. Pieces of stems showing disease symptoms were disinfected and subsequently placed on PDA medium. Fungal colonies emerging from plant material fragments were subcultured and subjected to further incubation. Fungi were identified by evaluating the morphological characteristics of colonies and macroconidia, based on available mycological keys. Discs were taken from the grown fungal colonies and placed onto potato dextrose agar (PDA) medium solidified in Petri dishes (90 mm). Biopreparations and the reference fungicide were added to the medium cooled to 45 °C in amounts allowing for the attainment of appropriate concentrations. The experiment was conducted in 5 replications.

The plates were incubated at 20 °C under controlled conditions in a Binder chamber (Sanyo Electric Japan—Incubator MIR-254, PHC Europe BV, Etten-Leur, The Netherlands). Incubation conditions were 20 °C for 14 h during the day and 14 °C for 10 h at night, at 50% humidity. When the mycelium had covered the surface of the medium in a given untreated object, the diameter of cultures in individual combinations was measured. For slow-growing cultures, measurements were taken after 3 weeks. The mean mycelial growth increment in millimeters and the percentage of mycelial growth inhibition were then calculated according to the formula. All experiments were conducted in 2 independent series.

In the greenhouse experiment, pot tests were conducted for winter wheat of the Turkis variety, aimed at evaluating germination, growth and development, and the biological utility of the tested bioproducts. In the greenhouse experiment, pot tests were conducted for winter wheat of the Turkis variety, aimed at evaluating germination, growth and development, and the biological utility of the tested bioproducts. The soil was inoculated before sowing. For inoculum preparation, *Fusarium* isolate (*F. culmorum*, *F. graminearum*, *F. poae*, *F. avenaceum*) was grown on PDA plates for 14 days at 22 ± 2 °C under continuous fluorescent light to promote sporulation. Conidial suspensions of the four *Fusarium* species were combined in equal proportions to create a polyspecies inoculum. The final mixed suspension was adjusted to a concentration of 1.0 × 10^6^ conidia mL^−1^. Experimental soil (sandy loam, pH 6.8) was collected from agricultural fields. Pre-sowing soil inoculation was performed by incorporating the standardized conidial suspension into soil at a rate of 50 mL suspension per kg of soil. The inoculated soil was thoroughly mixed and allowed to equilibrate for 24 h at room temperature (22 ± 2 °C) before sowing to ensure uniform pathogen distribution. Non-inoculated control treatments received soil mixed with sterile distilled water (50 mL kg^−1^ soil) to account for potential effects of soil moisture manipulation during the inoculation procedure. Wheat seeds were sown in the prepared soil. At the 4–5 leaf stage of wheat, the plants were treated by spraying with the tested biopreparations. Product doses are provided in Table 3.

All greenhouse experiments were conducted under controlled and continuously monitored environmental conditions throughout the experimental period: temperature 20 ± 2 °C, photoperiod 16/8 h (natural daylight supplemented with high-pressure sodium vapor lamps, 400 W), and relative humidity 65–75%. Climate parameters were recorded automatically by a digital environmental monitoring system to ensure uniform growing conditions across all treatments and replicates.

Comprehensive plant health assessment included both above-ground and below-ground biomass measurements. Greenhouse trials employed winter wheat with detailed morphometric analysis: (1) disease severity visual assessment (0–100 scale), (2) root length measurement from crown to longest tip, (3) shoot length from soil surface to highest leaf point, (4) shoot fresh weight of entire above-ground biomass, (5) root fresh weight of complete root system after careful washing, and (6) growth allocation efficiency calculated as shoot-to-root fresh weight ratio.

## 5. Conclusions

The present study demonstrates that garlic and Jerusalem artichoke extracts are rich sources of bioactive compounds with potential health-promoting properties. Garlic extract was particularly abundant in allicin, betulin, selected amino acids, and vitamin E, whereas polyphenols and some vitamins were present at lower levels. Garlic extract contained essential minerals, while heavy metals were negligible, confirming its safety for potential use. These properties suggest that the extracts have potential as natural biostimulants and eco-friendly crop protection agents, offering sustainable alternatives to synthetic fertilizers and pesticides in agriculture.

This study establishes a paradigm shift in biocontrol evaluation by demonstrating that comprehensive plant health assessment reveals additional value dimensions beyond traditional pathogen inhibition metrics. These findings fundamentally advance the scientific understanding of biological control mechanisms while providing practical tools for implementing sustainable wheat protection systems.

This research was funded by the Ferti-UP project supported by the National Centre for Research and Development (NCBiR), Poland, conducted in collaboration between Grupa Azoty Zakłady Azotowe “Puławy” S.A. and the Institute of Plant Protection—National Research Institute, Poznań.

## Figures and Tables

**Table 1:** Total phenol content in plant extracts (mean ± SD, *n* = 3).

No.	Product Name	Total Phenol Content (mg GAE/100 g of Extract)
1	Garlic (*Allium sativum*)	507.24 ± 12.35 ^a^
2	Jerusalem artichoke (*Helianthus tuberosus*)	595.68 ± 15.42 ^b^

Values followed by different superscript letters differ significantly at *p* < 0.05.

**Table 2:** Qualitative analysis of aqueous plant extracts.

Test	Garlic Extract	Jerusalem Artichoke Extract
Saponins (foam test)	+	−
Saponins (olive oil test)	+	+
Tannins	−	+
Terpenoids (Salkowski test)	−	−
Anthraquinones (Borntrager’s test)	−	−
Cardiac glycosides	−	−
Flavonoids	+	+
Carbohydrates	−	−
Coumarins	−	−
Phlobatannins	−	−

Note: (+) presence detected; (−) not detected.

**Table 3:** Abscisic acid (µg/mL) content in garlic extract (mean ± SD).

Compound	Concentration
Abscisic acid (ABA) (µg/mL)	6.45 ± 2.12

**Table 4:** Elemental composition of garlic extract (mean ± SD).

Element	Concentration (mg/L)
S	481.81 ± 12.0
Hg	0.00 ± 0.00
P	860.00 ± 21.5
Na	5.65 ± 0.15
K	2831.22 ± 70.8
Ca	348.46 ± 8.7
Mg	128.24 ± 3.2
Fe	4.99 ± 0.12
Al	1.05 ± 0.03
Ti	0.06 ± 0.002
Mn	2.74 ± 0.07
Cu	0.76 ± 0.02
Zn	6.69 ± 0.17
Cr	0.00 ± 0.00
Ni	0.00 ± 0.00
Pb	0.00 ± 0.00
V	0.00 ± 0.00
Zr	0.01 ± 0.0003
Co	0.00 ± 0.00
Li	0.01 ± 0.0003
Sr	0.92 ± 0.02
Ba	0.31 ± 0.01
As	0.03 ± 0.001
Cd	0.02 ± 0.001

**Table 5:** Contents of selected bioactive compounds in garlic extract.

Compound Category	Compound	Result ± SD (n = 2)	Unit
Allicin	Allicin	83.29 ± 0.34	mg/L
Free Amino Acids	L-Asparagine	10.44 ± 0.27	mg/L
	L-Aspartic acid	230 ± 3.51	mg/L
	Glycine	28.41 ± 0.84	mg/L
	L-Threonine	57.16 ± 0.11	mg/L
	L-Cysteine	<MQL (0.47)	mg/L
Triterpenes	Oleanolic acid	<MQL (7.60)	mg/L
	Maslinic acid	<MQL (5.50)	mg/L
	Lupeol	<MQL (6.30)	mg/L
	Betulin	275.10 ± 0.03	mg/L
Polyphenols	Gallic acid	<MQL (0.22)	mg/L
	Trans-caffeic acid	<MQL (0.20)	mg/L
	Quercetin	<MQL (0.83)	mg/L
	Rutin	<MQL (0.21)	mg/L
	(−)-Epicatechin	<MQL (0.21)	mg/L
	(+)-Catechin	<MQL (0.18)	mg/L
B Vitamins	Vitamin B2 (Riboflavin)	0.41 ± 0.01	mg/L
	Vitamin B3 (Niacin)	0.64 ± 0.02	mg/L
	Vitamin B6 (Pyridoxine)	1.06 ± 0.01	mg/L
	Vitamin B9 (Folic acid)	<MQL (0.05)	mg/L
Vitamin C	L-Ascorbic acid	<MQL (5.00)	mg/L
Vitamin A	Retinol	<MQL (5.00)	mg/L
Vitamin E	D-α-Tocotrienol	28.02 ± 2.18	mg/L
	D-β-Tocotrienol	<MQL (6.03)	mg/L
	D-γ-Tocotrienol	<MQL (5.15)	mg/L
	D-δ-Tocotrienol	<MQL (5.68)	mg/L
	D-α-Tocopherol	<MQL (5.49)	mg/L
	D-β-Tocopherol	<MQL (5.63)	mg/L
	D-γ-Tocopherol	24.29 ± 1.37	mg/L
	D-δ-Tocopherol	<MQL (5.87)	mg/L

Note: Values are mean ± standard deviation (n = 2) or below the method quantification limit (MQL).

**Table 6:** HPLC calibration curves for lactose, glucose, succinic acid, lactic acid, glycerol, acetic acid, 2,3-butanodiol, and ethanol.

Compounds	Equation	R^2^	Retention Time (min)
Succinic acid	y = 123,143x	0.999	14.63
Lactic acid	y = 115,258x	0.999	15.76
Glycerol	y = 123,143x	0.999	16.51
Acetic acid	y = 79,962.9x	0.999	18.36
2,3-Butanodiol	y = 148,757x	0.999	22.18
Ethanol	y = 63,562.6x	0.999	26.28

**Table 7:** Antifungal efficacy (% inhibition of mycelial growth) of biocontrol agents at different concentrations against wheat fungal pathogens.

Pathogen	Garlic Extract			*H. tuberosus Extract*			*Enterobacter* sp.			*Paenibacillus* sp.			Prothioconazole
	1%	5%	10%	1%	5%	10%	1%	5%	10%	1%	5%	10%	All conc.
*F. avenaceum*	16.3 *	7.8 *	2.2 *	37.8 *	39.4 *	34.1 *	59.1 *	49.1 *	31.3 *	74.1 *	45.4 *	60.0 *	100 ***
*F. culmorum*	5.6 *	31.5 *	46.7 *	37.6 *	53.1 *	74.6 *	0.0 ns	1.1 *	0.0 ns	50.7 *	49.3 *	67.2 *	100 ***
*F. fujikuroi*	10.4 *	27.0 *	35.7 *	46.5 *	49.1 *	44.4 *	32.8 *	53.5 *	26.1 *	64.3 *	51.5 *	51.9 *	100 ***
*F. graminearum*	7.8 *	24.8 *	38.0 *	59.1 *	66.5 *	67.4 *	7.2 *	3.0 *	0.0 ns	78.0 *	61.1 *	68.3 *	100 ***
*F. poae*	18.3 *	55.9 *	62.4 *	39.3 *	50.2 *	52.4 *	23.0 *	18.3 *	3.7 *	81.1 **	82.4 **	90.7 **	100 ***
*R. cerealis*	14.8 *	65.7 *	89.1 **	75.0 *	82.4 **	100.0 ***	32.4 *	35.7 *	0.0 ns	82.2 **	84.3 **	89.1 **	100 ***
*T. yallundae*	27.2 *	26.1 *	72.2 *	35.6 *	48.1 *	56.9 *	61.2 *	73.3 *	64.5 *	82.4 **	71.5 *	62.3 *	100 ***

Asterisks indicate values that are statistically significantly different from zero. *** *p* < 0.001 ** *p* < 0.01 * *p* < 0.05 (>0% inhibition—significant), ns = not significant.

**Table 8:** Disease severity of wheat and biometric parameters.

Treatment	Disease Severity (%)	Root Length (cm)	Shoot Length (cm)	Shoot Fresh Weight (g)	Root Fresh Weight (g)	Total Biomass (g)	Growth Efficiency	Significance
Garlic extract	63.1 ± 7.1	20.7 ± 1.2	43.3 ± 2.4	32.7 ± 3.1	5.2 ± 1.1 **	37.9 ± 3.8	6.3 ± 1.2	*p* < 0.01
*H. tuberosus*	43.6 ± 12.6 **	19.6 ± 1.1	43.7 ± 2.0 *	36.9 ± 9.9 *	5.2 ± 1.3 **	42.1 ± 10.8 **	7.1 ± 1.8 *	*p* < 0.01
*Enterobacter*	44.6 ± 13.7 **	19.9 ± 1.5	43.0 ± 1.5	33.6 ± 5.9	4.7 ± 0.6 ***	38.3 ± 6.2	7.1 ± 1.4 *	*p* < 0.001
*Paenibacillus*	65.0 ± 15.7	20.3 ± 1.9	40.2 ± 4.6	29.7 ± 5.2	5.0 ± 0.6 **	34.7 ± 5.5	5.9 ± 1.3	*p* < 0.01
Prothioconazole	14.5 ± 9.5 ***	22.4 ± 2.8 *	42.6 ± 4.3	38.2 ± 5.4 **	6.7 ± 2.9	44.9 ± 7.8 ***	5.7 ± 2.1	*p* < 0.001
Untreated control	66.4 ± 6.8	22.0 ± 2.9	38.6 ± 4.7	31.5 ± 2.9	9.1 ± 3.2	40.6 ± 5.8	3.5 ± 1.1	—

* *p* < 0.05, ** *p* < 0.01, *** *p* < 0.001 Growth Efficiency = Shoot Fresh Weight/Root Fresh Weight ratio.

**Table 9:** Characteristics of the natural products used in in vitro experiments and greenhouse studies.

No.	Product Name	Concentration (After Dilution of Initial Extract) % (*w*/*v*) In Vitro	Selected Concentration in Greenhouse Studies % (*w*/*v*)	Additional Data
1	Garlic (*Allium sativum*)	1, 5, 10	10	water extract of garlic (bulb), DER 1:2.5; ultrasound-assisted extraction; conditions: time of extraction: 1 h, temperature: 60 °C
2	Jerusalem artichoke (*Helianthus tuberosus*)	1, 5, 10	10	water extract of Jerusalem artichoke (leaves), DER 1:9; ultrasound-assisted extraction; conditions: time of extraction: 1 h, temperature: 60 °C
3	*Enterobacter* supernatant	1, 5, 10	1	fermentation broth (GAP1) obtained by the *Enterobacter* bacteria in a bioreactor
4	*Paenibacillus* supernatant	1, 5, 10	1	fermentation broth (372) obtained by the *Paenibacillus* bacteria in a bioreactor
5	Prothioconazole	0.05 L	0.05 L	Protikon 250 EC (250 g active ingredient/L)

**Table 10:** Characteristics of the pathogens used in the in vitro experiment.

No.	Pathogen	Isolate No.
1	*Fusarium avenaceum*	7A/2022
2	*Fusarium culmorum*	33B/2021
3	*Fusarium fujikuroi*	14D/2021
4	*Fusarium graminearum*	10A/2022
5	*Fusarium poae*	11A/2021
6	*Rhizoctonia cerealis*	23B/2023
7	*Tapesia yallundae*	14A/2022

## Data Availability

The original contributions presented in this study are included in the article. Further inquiries can be directed to the corresponding author.
